# Repeatability and Reproducibility of Total Retinal Blood Flow Measurements Using Bi-Directional Doppler OCT

**DOI:** 10.1167/tvst.9.7.34

**Published:** 2020-06-25

**Authors:** Stephan Szegedi, Nikolaus Hommer, Martin Kallab, Stefan Puchner, Doreen Schmidl, René M. Werkmeister, Gerhard Garhöfer, Leopold Schmetterer

**Affiliations:** 1 Department of Clinical Pharmacology, Medical University of Vienna, Austria; 2 Center for Medical Physics and Biomedical Engineering, Medical University of Vienna, Austria; 3 Singapore Eye Research Institute, Singapore National Eye Centre, Singapore; 4 School of Chemical and Biomedical Engineering, Nanyang Technological University, Singapore; 5 SERI-NTU Advanced Ocular Engineering (STANCE), Singapore; 6 Ophthalmology and Visual Sciences Academic Clinical Program, Duke-NUS Medical School, Singapore; 7 Institute of Clinical and Experimental Ophthalmology, Basel, Switzerland

**Keywords:** retinal blood flow, healthy subjects, repeatability

## Abstract

**Purpose:**

To investigate the repeatability and reproducibility of total retinal blood flow measurements using a custom-built dual-beam bidirectional Doppler optical coherence tomography (OCT) system in healthy subjects.

**Methods:**

Repeatability and reproducibility were analyzed in 10 and 34 healthy subjects, respectively. For repeatability, measurements were taken twice within 30 minutes, for reproducibility, twice within two to five weeks. Two analysis approaches were compared for calculation of absolute blood velocities: a previously published approach resulting in values for total arterial (Q_A,abs_) and total venous blood flow (Q_V,abs_) and a novel approach taking into account that there is a fixed relation between the phase shift in the two OCT channels (Q_A,new,_ Q_V,new_). Repeatability and reproducibility were quantified using intraclass correlation coefficients (ICCs).

**Results:**

For Q_A,abs_ and Q_V,abs_, ICC values between 0.78 and 0.84 were obtained. Q_A,new_ and Q_V,new_ values revealed better repeatability and reproducibility as compared to the convential appoach. Repeatability ICCs for Q_A,new_ and Q_V,new_ were between 0.91 and 0.93, and reproducibility ICCs were between 0.87 and 0.91 indicating excellent reproducibility. Good agreement was observed between total retinal blood flow values as measured from retinal arteries and retinal veins.

**Conclusions:**

Measurement of total retinal blood flow using dual-beam Doppler OCT shows excellent reproducibility, which can further be improved by using a novel algorithm for calculating blood velocities in retinal vessels.

**Translational Relevance:**

Our data indicate that dual-beam Doppler OCT can be used for longitudinal studies. Hence, quantitative retinal blood flow may be established as a biomarker for progression vascular eye diseases.

## Introduction

Alterations of ocular perfusion parameters have been described in a wide variety of ocular and systemic diseases.^1^^–^^4^ In retinal occlusive diseases, the degree of ischemia is directly related to the visual outcome.^5^^,^^6^ In diabetic retinopathy, alterations in retinal perfusion and changes in the retinal vasculature are observed early in the disease process, but the significance concerning the pathophysiology of the disease is currently incompletely understood.^4^^,^^7^^–^^10^ In glaucoma, retinal perfusion changes are unequivocally shown, but it is not fully established whether these are cause or consequence of the disease.^11^^–^^13^

Optical coherence tomography (OCT) angiography has recently become commercially available for imaging the ocular microvasculature,^14^^–^^16^ but it has the inherent limitation that perfusion cannot be quantified. Other techniques for measurement of blood flow such as laser Doppler velocimetry^17^^,^^18^ or laser Doppler flowmetry^19^^,^^20^ have been introduced, but because of the demanding signal acquisition, these imaging modalities are not used in clinical practice. Laser speckle flowgraphy (LSFG) is now commercially available and has been used to study the retinal circulation,^21^^–^^23^ but the values obtained are not absolute.

Doppler OCT is a functional extension of OCT capable of measuring absolute blood flow in the retina.^24^ Whereas the technology is not yet commercially available, prototype systems have been developed and repeatability and reproducibility have been reported.^25^ We have built a dual-beam bidirectional Doppler OCT system^26^ that is capable of measuring total retinal blood flow.^27^^,^^28^ In the present study, we set out to evaluate the repeatability and reproducibility of this technology in healthy subjects and also evaluated different methods for velocity analysis. We previously used an algorithm for calculating retinal blood velocities based on the geometrical setup of the optical system.^29^ We have, however, recently shown that there is an alternative way of calculating absolute blood velocities by taking into account that there is a fixed angle between the two incident beams.^26^ As such an additional aim of this study was to compare the two algorithms.

## Research Design and Methods

### Subjects

The study protocol was approved by the Ethics Committee of the Medical University of Vienna and followed the guidelines of the Declaration of Helsinki. We included a total of 34 healthy volunteers aged between 18 and 39 years. Written informed consent was obtained from all participating subjects. All participating subjects passed a screening examination that included physical examination, visual acuity testing, biomicroscopy, funduscopy and intraocular pressure (IOP) measurement using Goldmann applanation tonometry. Inclusion criteria were visual acuity ≥ 20/20, IOP < 21 mm Hg and normal findings in the ophthalmic examination. Subjects had to be nonsmokers to be included. Exclusion criteria were ametropia ≥ 3 diopters, anisometropia ≥ 3 diopters, presence of systemic disease, blood donation, or intake of any medication in the three weeks before the study and any clinically relevant abnormalities found in the screening visit as judged by the investigators. Participants were asked to abstain from alcohol- or caffeine-containing beverages for at least 12 hours before the study visit.

### Protocol

In all subjects the right eye was chosen for the measurements. The measurements were performed in a dark room at pleasant room temperature. The pupil was dilated using topical administration of tropicamide (Mydriatikum AGEPHA, Vienna, Austria). Thereafter, a resting period of at least 20 minutes was scheduled. This was done to achieve full mydriasis and stabilize blood pressure and pulse rate, which was verified by repeated measurements. On each study day measurement of blood pressure, pulse rate, and IOP was done before blood flow measurements. A fundus photograph was obtained where all measurement points were marked to ensure that measurements were performed at the same vessel position each time. Retinal blood flow was measured using a previously described custom-built dual-beam bidirectional Doppler Fourier-domain OCT.^27^ Each measurement of total retinal blood flow took approximately 10 to 15 minutes for each individual.

Repeatability was tested in a subgroup of 10 healthy subjects. For this purpose, the Doppler OCT acquisition was repeated twice within 30 minutes. Reproducibility was tested in the entire group of 34 healthy subjects. The calculation was based on measurements that were obtained two to five weeks apart.

### Measurement of Retinal Blood Flow

**Figure 1.**
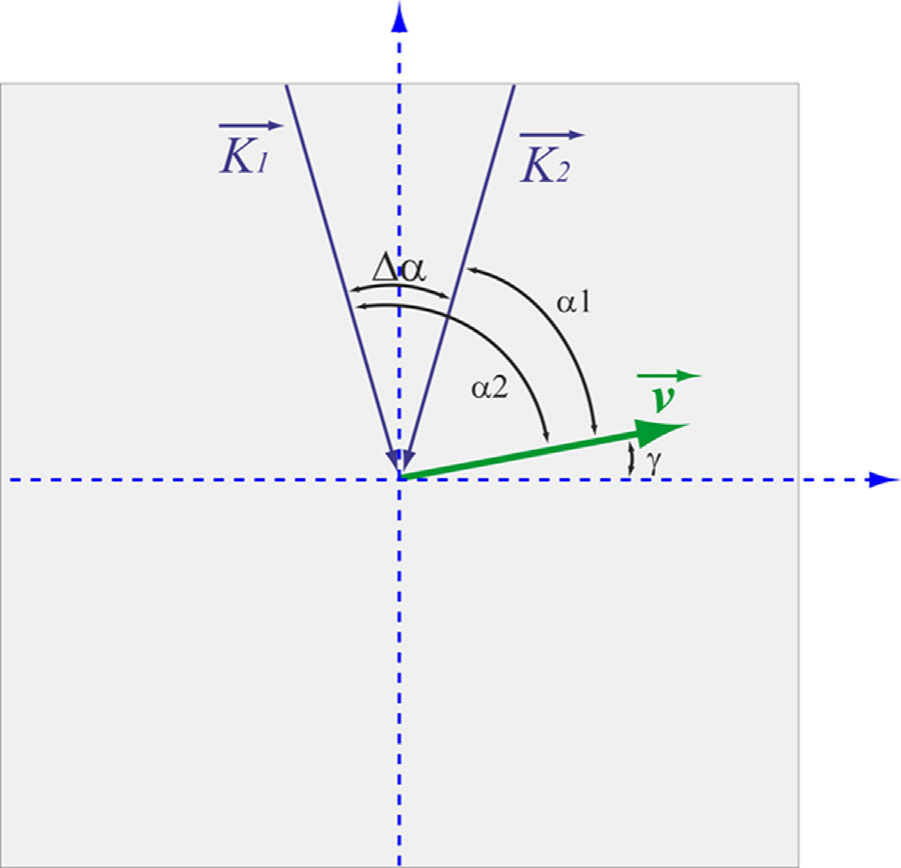
Geometrical setting at the posterior pole of the eye. ${\vec{K}}_{1,2}$, probe beam wave vectors; $\vec{v}$, velocity vector; α_1/2_, Doppler angles; Δα, separation angle between probe beams; γ, angle between $\vec{v}$ and the plane perpendicular to the detection plane.

As mentioned above, a custom-built dual-beam Doppler Fourier-domain OCT system coupled to a fundus camera was used for the measurements. Measurements were performed in all retinal arteries and veins with a diameter of at least 40 µm. To ensure that each vessel is captured, a specific rectangular scanning pattern centered around the optic nerve head was used.^27^ The scanning of each area was done once and lasted several seconds to allow for averaging of blood velocity values over several pulse periods. The system and analysis of data have been described in a number of previous publications and will therefore only be summarized shortly here.^7^^,^^26^^,^^27^^,^^30^^–^^32^ The technology is based on the illumination of the target tissue with two laser beams separated by an angle Δα being the difference between the Doppler angles α_i_ of the two measurement channels (Δα = α_1_ – α_2_). Hence, given by the different Doppler angles, the phase changes that are detected in the two detection units are different. We have previously reported that absolute blood velocity can be measured with this geometric arrangement (Fig. 1) if the angle Δα between the beams is known:^26^^,^^29^^,^^30^

(1) $$V_{abs}=\Delta \Phi \frac{\lambda }{4\pi \cdot n\cdot \tau \cdot \Delta \alpha \cdot \cos\beta }.$$

In this equation, ΔΦ is the difference in the phase shift between channel 1 (Φ_1_) and channel 2 (Φ_2_). The λ is the central wavelength of the OCT light source,  τ is the time interval between two subsequent recordings that is dependent of the acquisition rate of the system, n is the group refractive index of blood, and β is the angle between the plane spanned from the two incoming laser beams and the velocity vector, which can be determined based on the fundus image or the en face projection of the OCT image data.

In this study we have used a novel approach to calculate the absolute blood velocity.

The phase shifts in the two channels can be written as follows:^26^

(2) $$\Phi_{1}=V_{abs}\cdot \left(\frac{4\pi n\tau cos\beta }{\lambda }\right)\cdot \left(\frac{\pi }{2}-ɛ\right)$$

(3) $$\Phi_{2}=V_{abs}\cdot \left(\frac{4\pi n\tau cos\beta }{\lambda }\right)\cdot \left(\frac{\pi }{2}-ɛ-\Delta \alpha \right)$$

In these equations, the angle ε is given by α_1_− γ, where γ is the angle between the velocity vector and the plane perpendicular to the optical axis of the illuminating beams. When V_abs_ is calculated based on Equation 1, two values for ε, which we call ε_1_ and ε_2_, can be calculated based on Equations 2 and 3, respectively. Thereafter, using Equation 1 and the angles ε_1_ and ε_2_ the absolute velocity values V_abs,1_ and V_abs,2_ can be obtained for each channel separately. We have previously used these calculations for checking the quality of our measurements. In this study we used a different approach and calculated V_new_ as (V_abs,1_ + V_abs,2_)/2.

To calculate the volumetric blood flow, the vessel's diameter (d) needs to be extracted. We have previously developed an approach for measuring vessel caliber using the phase image.^33^ In the current study, blood flow in retinal vessels was calculated using our previous approach as Q_abs_ = V_abs_ d^2^ π/4 and the novel approach Q_new_ = V_new_ d^2^ π/4. Total retinal blood flow was either calculated by summing up all data from retinal arteries (Q_A,abs_, Q_A,new_) or all data from retinal veins (Q_V,abs_, Q_V,new_).

### Measurement of Intraocular Pressure, Blood Pressure, and Pulse Rate

Measurement of IOP was done using a slit-lamp mounted Goldman applanation tonometer. Automated oscillometry (Infinity Delta; Dräger, Vienna, Austria) on the upper arm was used to measure systolic, diastolic, and mean arterial blood pressures (systolic blood pressure [SBP], diastolic blood pressure [DBP], and mean arterial pressure [MAP]) and pulse rate. As proposed previously, ocular perfusion pressure (OPP) in the sitting position was calculated as OPP = 2/3*MAP-IOP^34^ taking into account the pressure drop between the upper arm and the eye.

### Data Analysis

The statistical analysis of the data was performed using CSS Statistica (Release 6.0; StatSoft Inc., Tulsa, OK, USA). A Shapiro–Wilk test was performed to test the data for normal distribution. Reproducibility was calculated using the data obtained within 30 minutes. Repeatability was quantified based on the data obtained within two weeks. The two obtained measurements were compared using paired *t* tests. To quantify reliability of measurements, intraclass correlation coefficients (ICC) were calculated. ICC values less than 0.5, between 0.5 and 0.75, between 0.75 and 0.90, and greater than 0.90 represent poor, moderate, good, and excellent repeatability, respectively.^35^ In addition, the coefficient of variation (CoV) was calculated, and the Bland-Altman plot was drawn. Linear correlation analysis was performed to investigate whether age, sex, and ocular perfusion pressure were associated with total retinal blood flow. Data are presented as means ± SD. A *P* value < 0.05 was considered the level of significance.

## Results

### Repeatability

**Figure 2.**
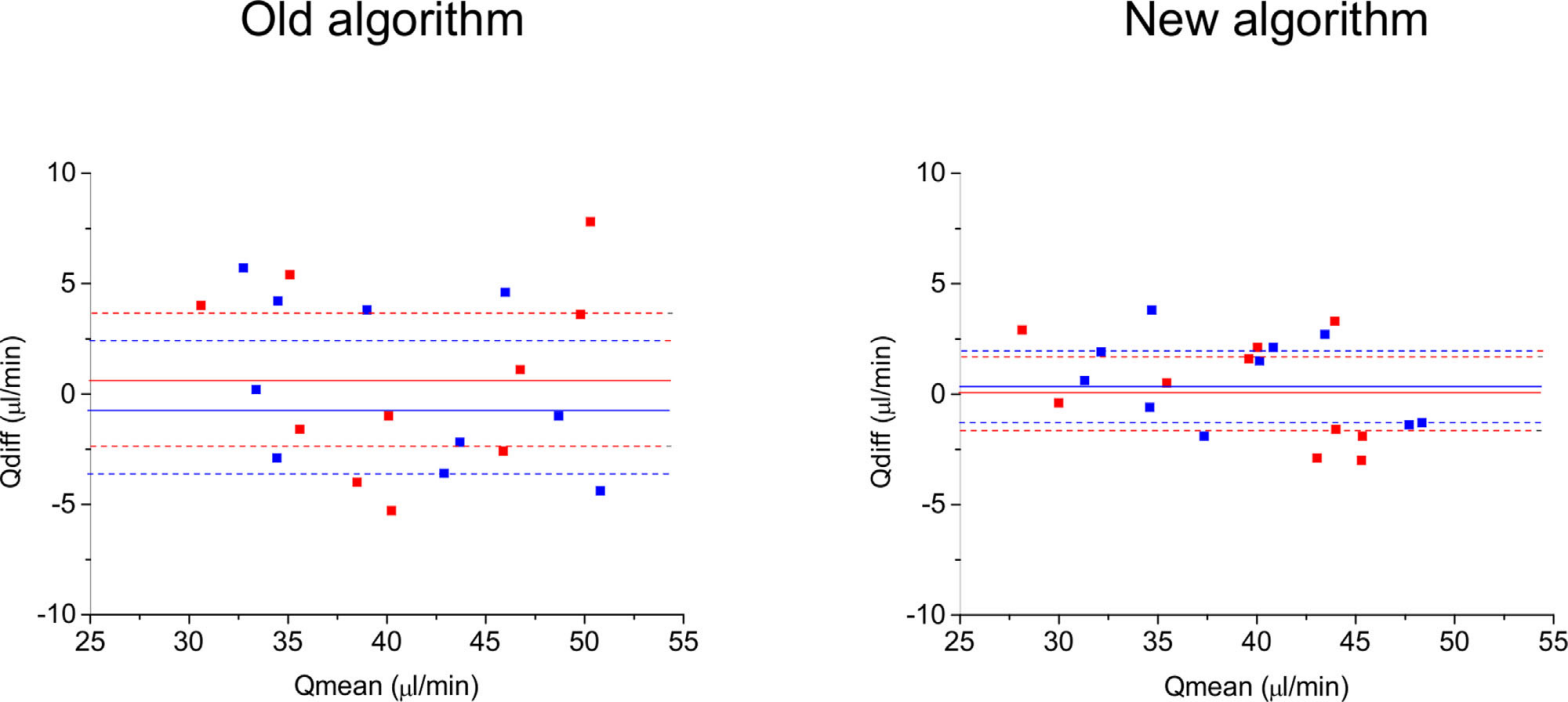
Bland-Altman plots for repeatability data. The x-axis shows the mean between the two measurements (Qmean), the y-axis shows the difference between the two measurements (Qdiff). The data for arteries are presented in red and the data for veins are presented in blue. The mean value of the difference (solid line) and the 95% confidence intervals are also shown (dashed line).

The mean age of the participating subjects was 24 ± 5 years (five female, five male). All outcome parameters were normally distributed. The subjects had normal blood pressure with average values of 122 ± 7 mm Hg, 59 ± 5 mm Hg and 82 ± 6 mm Hg for SBP, DBP, and MAP, respectively. With an IOP of 14 ± 2 mm Hg, the calculated OPP was 42 ± 4 mm Hg. The values obtained for total retinal blood flow are presented in Table 1. Total retinal blood flow was not correlated to age, sex, or OPP (data not shown). Data quantified from arteries (Q_A,abs_) and veins (Q_V,abs_) using our previous published method were not significantly different (*P* = 0.321), indicating good consistency of measurements. When the data were processed with the novel algorithm, the difference between results obtained from arteries (Q_A,new_) and veins (Q_V,new_) was even lower (*P* = 0.631). The ICCs and CoVs are also presented in Table 1. Whereas the repeatability was good for Q_A,abs_ and Q_V,abs_, the ICC values improved to be excellent for Q_A,new_ and Q_V,new_. The Bland-Altman plot for repeatability data is depicted in Figure 2. The flow values as obtained using the new algorithm were lower than those with our previous algorithm (arteries: *P* = 0.021, difference −1.7 ± 1.9 µL/min; veins: *P* = 0.012, difference −1.6 ± 1.6 µL/min), but the difference was less than 5%.

**Table 1.** Total Retinal Blood Flow as Obtained From Retinal Arteries and Veins Using Our Previous Algorithm (Q_A,Abs_ and Q_V,Abs_) and Our New Algorithm (Q_A,New_ and Q_V,New_) as Obtained in the Repeatability Cohort (n = 10)

	Values (µL/min)	Intraclass Correlation Coefficient	Coefficient of Variation (%)
Q_A,abs_	41.3 ± 6.8	0.81	6.3 ± 3.7
Q_V,abs_	40.6 ± 6.4	0.84	5.8 ± 3.4
Q_A,new_	39.5 ± 6.2	0.91	3.8 ± 2.3
Q_v,new_	39.0 ± 5.9	0.93	3.3 ± 1.9

All data are the mean of the two measurements. Data are presented as means ± SD.

### Reproducibility

**Figure 3.**
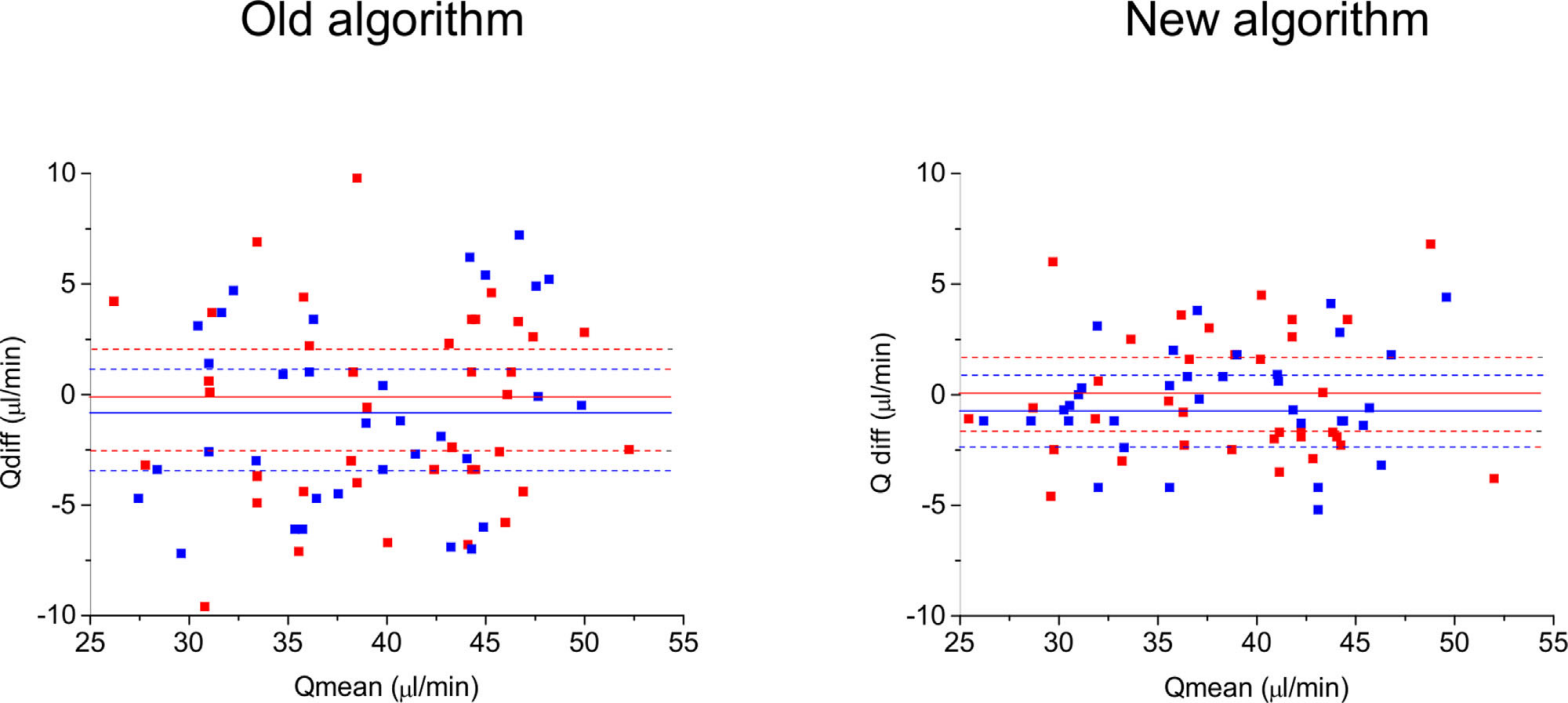
Bland-Altman plots for reproducibility data. The x-axis shows the mean between the two measurements (Qmean), the y-axis shows the difference between the two measurements (Qdiff). The data for arteries are presented in red and the data for veins are presented in blue. The mean value of the difference (solid line) and the 95% confidence intervals are also shown (dashed line).

The mean age of the subjects who participated in the reproducibility experiments was 25 ± 5 years. Again, an equal number of female and male subjects were included (n = 17 each). The blood pressure values were in the same range as those in the repeatability cohort (day 1: SBP: 119 ± 6 mm Hg, DBP: 57 ± 4 mm Hg and MAP: 79 ± 6 mm Hg, day 2: 120 ± 6 mm Hg, DBP: 58 ± 5 mm Hg and MAP: 80 ± 6 mm Hg). Because the IOP was normal in the participating subjects (day 1: IOP: 14 ± 3 mm Hg, day 2: IOP: 14 ± 3 mm Hg), the OPP was also in the normal range (day 1: 38.2 ± 3.9 mm Hg, day 2: 38.7 ± 4.0 mm Hg). In keeping with our results in the repeatability experiments, total retinal blood flow values were not correlated to age, sex, or OPP (data not shown). The total retinal blood flow data are provided in Table 2. Using our previous published method, total retinal blood flow as measured from arteries (Q_A,abs_) was slightly higher than those obtained from retinal veins (Q_V,abs_, *P* = 0.043). This difference between arterial and venous data was not seen when the novel algorithm was used for analysis (Q_A,new_, Q_V,new_; *P* = 0.165). As expected, ICCs for reproducibility were slightly worse than those for repeatability. The obtained values indicate good reproducibility for Q_A,abs,_ Q_V,abs_ and Q_A,new_, and excellent reproducibility for Q_V,new._ Figure 3 shows the Bland-Altman plot for reproducibility data. In keeping with our results obtained in the repeatability measurements, values obtained using the novel algorithm in retinal veins were slightly lower than those obtained with our previous algorithm in retinal veins (*P* = 0.024, difference −1.7 ± 4.1 µL/min). By contrast, Q_V,abs_ and Q_V,new_ were not significantly different (*P* = 0.087, difference −1.1 ± 3.9 µL/min).

**Table 2.** Total Retinal Blood Flow as Obtained From Retinal Arteries and Veins Using Our Previous Algorithm (Q_A,Abs_ and Q_V,Abs_) and Our New Algorithm (Q_A,New_ and Q_V,New_) as Obtained in the Reproducibility Cohort (n = 34)

	Values (µL/min)	Intraclass correlation coefficient	Coefficient of variation (%)
Q_A,abs_	40.1 ± 6.5	0.78	6.7 ± 5.1
Q_V,abs_	39.4 ± 6.3	0.79	7.0 ± 4.3
Q_A,new_	38.4 ± 6.0	0.87	4.5 ± 2.9
Q_v,new_	38.1 ± 6.2	0.91	3.4 ± 2.6

All data are the mean of the two measurements. Data are presented as means ± SD.

## Discussion

The present study evaluated the repeatability and reproducibility of total retinal blood flow measurements in healthy subjects using dual-beam bidirectional Doppler OCT. Two major results were found: 1. The repeatability and reproducibility of the Doppler OCT system is good to excellent and should be sufficient to be used in longitudinal studies. 2. The variability of data can be further reduced by using a novel algorithm for the calculation of absolute blood velocity.

Published data on the reproducibility of quantitative retinal blood flow measurements are scarce. Repeatability and reproducibility of our measurements were slightly lower than those performed with a commercial Doppler OCT prototype, where the intraclass correlation coefficients exceeded 0.90 for both retinal arterioles and venules.^25^ In the latter study, however, blood flow was only measured in one large retinal artery and one large retinal vein. By contrast, our system measures absolute blood flow in all vessels including vessels as small as 40 µm, which is a requirement to obtain total retinal blood flow. This can well explain the differences in repeatability and reproducibility between the two approaches. In comparison to laser Doppler velocimetry,^36^ the reproducibility appears to be improved with Doppler OCT, which may facilitate clinical application.

Approaches other than dual-beam OCT have been developed to measure total retinal blood flow. This includes three-beam systems,^37^^–^^39^ scans obtained in a pattern of two concentric circles,^40^^–^^42^ as well as en face approaches.^43^^–^^45^ Reproducibility data have only been provided for data obtained in a concentric circle pattern and are in the same order as in this study.^46^ We have previously presented a method for calculating total retinal oxygen extraction by coupling the dual-beam Doppler OCT system to a fundus camera.^7^^,^^31^^,^^32^ Although we have not explicitly studied the repeatability and reproducibility of this approach, it can be estimated based on our previous results on the variability of oxygen saturation measurements^47^ and the data obtained in this study.

In this paper we introduce a novel method for obtaining absolute velocity data. The results of this study indicate that this approach shows higher reproducibility than our previous algorithm.^26^^,^^30^ The novel algorithm takes advantage of the fact that there is a given relation between the velocities as measured in the two channels related to the angle between the two laser beams. Hence, the novel algorithm is less affected by measurement errors in one of the two channels. The absolute blood flow values as obtained with this approach are slightly lower, but because the difference is small, we do not deem it problematic in applying the novel approach. The reason for this difference is currently unknown and requires further investigation.

Total retinal blood flow values as obtained in this study were neither correlated with age, sex, or OPP. Previous studies have indicated an age-related decline in retinal blood flow.^32^^,^^48^^,^^49^ In this study we did, however, include only healthy young subjects, and the age range was narrow. Whereas some studies indicate higher retinal blood flow in women,^50^ data are controversial, and no clear evidence for gender differences exists.^51^ The lack of correlation with OPP is in agreement with previous studies^52^^,^^53^ and is also expected in a highly autoregulated vascular bed.^54^

The present study has several limitations. Most importantly, we studied healthy subjects only. Thus we cannot exclude that reproducibility may be worse in patients with ocular disease, particularly when fixation abilities are affected. In addition, we did not study the long-term reproducibility of retinal blood flow as assessed with our prototype. Fluctuations of retinal blood flow may have two principal components: one related to the measurement error and one related to physiological changes such as blood pressure, heart rate, arterial blood gases, hormonal factors, and others that may occur over time. It is currently unknown how much retinal blood flow changes when these factors fluctuate, but the common view is that perfusion rate in the retina stays relatively constant as it does in the brain.^54^^,^^55^ However, considering the natural fluctuation of blood flow, our results may rather underestimate the long-term reproducibility of the system.

In conclusion, we have shown that measurement of total retinal blood flow shows excellent repeatability and reproducibility using a customized dual-beam Doppler OCT system in healthy subjects. This was achieved by using a novel algorithm for calculation of absolute blood velocities in healthy subjects. Our data indicate that the technique can be used in longitudinal studies assessing changes of retinal blood flow in patients.
